# P2X7: a receptor with a split personality that raises new hopes for anti-cancer therapy

**DOI:** 10.1007/s11302-021-09783-w

**Published:** 2021-04-05

**Authors:** Alba Clara Sarti, Valentina Vultaggio-Poma, Francesco Di Virgilio

**Affiliations:** Department of Medical Sciences, University of Ferrara, Ferrara, Italy

In a recent paper, Valerie Vouret-Craviari and coworkers show that controlled activation of the P2X7 receptor (P2X7R) inhibits growth of non-small cell lung cancer (NSCLC), promotes tumor regression in association with anti-PD-1 antibodies, and confers long-lasting CD8^+^ lymphocyte-mediated immunity [1]. The pathophysiological function of the P2X7 receptor (P2X7R) is still a puzzle 25 years after its cloning [2] and even longer since its role in immunity and cancer was first postulated [3] [4] [5]. For almost 3 decades, investigators’ attention focused on the peculiar property of this receptor to trigger cytotoxicity, by necrosis, apoptosis, or pyroptosis, depending on the given cell type and the experimental conditions [6]. The (apparently) unavoidable association between P2X7R gating and cell death depends on the peculiar ability of this receptor to open a non-selective plasma membrane pathway (the so-called macropore, whether intrinsic to the receptor or simply activated by the receptor is not clear), allowing uncontrolled fluxes of aqueous solutes across the plasma membrane [7]. However, it was clear from the very beginning of the P2X7R saga that there was more to this receptor than just “cytotoxicity” since anecdotal evidence suggested that its controlled activation (i.e., under conditions that do not trigger opening of the large conductance pore), far from causing cell death was, on the contrary, “beneficial” and associated with cytokine release [8], transcription factor stimulation [9], or even proliferation [10]. Due to the consolidated finding that cancer cells express high P2X7R levels [11], it was proposed that targeting this receptor with suitable selective blockers could be a viable approach for cancer therapy [12].

Tumors can evade the immune response by exploiting inhibitory immune check-points, such as the programmed cell death-1 (PD-1)/programmed cell death ligand-1 (PD-L1) pathway [13]. The introduction of immune check-point blockers in cancer therapy has raised high hopes. However, these innovative therapies are effective only in a small percentage of cases, and even responsive patients often relapse after a few months [13]. Thus, additional alternative approaches are urgently needed. The P2X7R has enjoyed wide interest as a potential new target for different and somehow opposite reasons. On one side, it has been proposed that its growth-promoting activity could be exploited, and thus that we could take advantage of the multiple and well-known highly selective P2X7R inhibitors developed by pharma industry [14]. On the other, it was suggested that P2X7R cytotoxic activity to kill tumors could be exploited, presumably sparing healthy cells, thanks to their lower level of P2X7R expression [15]. Overall, small molecule P2X7R blockers have proven to be effective as in vivo anti-cancer drugs [16], but results were not always very impressive, thus generating the general feeling that there should be better and more efficient ways to take advantage of the P2X7R for cancer therapy. Previous studies showed that, besides tumor cell-expressed P2X7R, immune cell-expressed P2X7R is also very important in anti-tumor response [17]. Thus, in principle, it should be possible to potentiate the anti-tumor immune response via P2X7R stimulation. But, how?

The P2X7R of immune cells is needed for several immune-related responses, such as chemotaxis, cytokine release, and antigen presentation [18]. Thus, an issue was raised on the use of P2X7R blockers as anti-cancer agents, which on one hand might no doubt inhibit tumor growth but on the other might also impair the anti-cancer immune response. This concern seems to be ruled out by the recent demonstration by De Marchi et al. that pharmacological manipulation of the P2X7R may substantially change immune cell tumor infiltration to the host’s advantage, as administration of a P2X7R blocker to wild-type mice promoted an increase in CD4^+^ effector cells, but left unaltered the number of CD8^+^ and Treg cells [19]. Now, the key burning current question is whether it is possible to combine P2X7R-targeting with procedures that take advantage of the unique purinergic signature of the tumor microenvironment (TME), i.e., the unusually high extracellular ATP (eATP) concentration.

The finding that eATP is an abundant constituent of the TME [20] is perhaps one of the most important recent discoveries in cancer biology, as it identified a highly selective biochemical marker of the TME, only shared by highly inflamed tissues. The demonstration of the therapeutic opportunities afforded by this finding is provided by the recent development of therapeutic antibodies that promote a striking activation of the immune system only in the TME, thus causing an efficient anti-cancer response in the absence of unwanted extra-tumor side effects [21] [22]. The trick to achieving tumor selectivity was to engineer an antibody that binds the cognate target (the CD137 receptor of NK cells) only in the presence of near-millimolar eATP concentrations. Valerie Vouret-Craviari and her coworkers in their recent paper took a further step forward by exploiting eATP in the TME together with P2X7R pharmacology [1].

One of the distinct pharmacological features of the P2X7R is its absolute ATP selectivity: this receptor is very faithful, since no physiological ligands beside eATP are known [23]. However, several positive allosteric modulators, i.e., molecules that activate the P2X7R only in the presence of eATP, presumably acting at sites different from the nucleotide-binding site, have been identified over the years [24] [25]. Thus, Vouret-Craviari and coworkers had the clever idea of verifying whether a novel P2X7R-positive allosteric modulator (HEI3090) might trigger a controlled activation of the P2X7R in the high eATP TME and thus achieve tumor elimination [1]. To set up a more thorough anti-cancer strategy, these authors combined HEI3090 treatment with αPD-1 immune check-point antibodies. Results were striking: 80% of mice (13 out of 16) inoculated with Lewis lung carcinoma (LLC), followed by the combined treatment (HEI3090 plus αPD-1), were tumor-free 20 days after tumor inoculation. On the contrary, only 6% (1 out of 16) was free of tumor in the cohort inoculated with αPD-1 alone. Even more strikingly, only the combined treatment allowed long-lasting survival (340 d). The combined treatment was also effective in reducing growth of B16-F10 melanomas and KRAS-driven lung cancer.

As pointed out above, both immune and cancer cells express the P2X7R at high levels; thus, the anti-cancer effect of HEI3090 might be due to activation of immune cell P2X7R (thus promoting release of immunostimulatory cytokines) or cancer cell P2X7R (thus triggering a cytotoxic response). However, experiments performed in *p2rx7*^*−/−*^ mice clearly ruled out an effect mediated by tumor-expressed P2X7R, since LLC tumor growth was unaffected by HEI3090 treatment. Thus, the relevant target of HEI3090 stimulation was immune cell P2X7R. The authors went further in dissecting the underlying mechanism by showing that controlled stimulation of dendritic cell P2X7R triggered the release of IL-18 (but curiously not of IL-1β) that in turn stimulated anti-tumor activity of NK and CD4^+^ lymphocytes. Importantly, the combined HEI3090 and αPD-1 treatment conferred a long-term anti-tumor memory immune response that protected from rechallenge with a subsequent inoculum with LLC cells. Finally, analysis of data from patients affected by NSCLC showed that high levels of *P2RX7* expression correlated with a high immune response.

The P2X7R is a dual-function ion channel that allows trans-membrane fluxes of mono- and di-valent cations, as well as the unrestricted passage of aqueous solutes of molecular mass up to 900 Da [7]. While small cation flux is in principle associated with the activation of trophic functions, unrestricted passage of larger solutes dramatically upsets intracellular ion balance and initiates a death (necrosis, apoptosis, or pyroptosis) pathway. Indirect evidence suggests that the P2X7R might undergo a “controlled” activation, leading to opening of the ion channel, but not of the large conductance pore, but until the report of Vouret-Craviari and coworkers [1], there was no in vivo evidence that pharmacological treatment could achieve this goal. Some hints of controlled P2X7R activation come from the discovery of P2X7R-positive allosteric modulators [24] [25]. Several compounds, whether natural or synthesized, synergize with eATP at the P2X7R in vitro. Many of these are drugs already approved by regulatory agencies for the treatment of various diseases. Administration of P2X7R-positive allosteric modulators might allow safe P2X7R gating only in those anatomical sites where eATP is elevated, e.g., at sites of cancer or strong inflammation, but not in healthy tissues.

Besides these exciting data providing the proof that the P2X7R may undergo controlled activation in vivo, the study by Vouret-Craviari and coworkers raises an intriguing question: why is the tumor itself not sensitive to the potentially cytotoxic effect due to P2X7R activation? At least two educated guesses are possible: (a) synergistic activation *per se* is not sufficient to cause an uncontrolled opening of the “macropore” and thus trigger cell death, and (b) cancer cell expresses a “non-functional” P2X7R. Whichever the case, these experiments suggest that it is possible to selectively stimulate the P2X7R of immune cells to potentiate the anti-tumor immune response in the absence of contextual stimulation of cancer P2X7R and of unwanted extra-tumor side effects.
